# Advances in the Management of HPV-Related Oropharyngeal Cancer

**DOI:** 10.1155/2019/9173729

**Published:** 2019-04-14

**Authors:** F. De Felice, V. Tombolini, V. Valentini, M. de Vincentiis, S. Mezi, O. Brugnoletti, A. Polimeni

**Affiliations:** ^1^Department of Radiotherapy, Policlinico Umberto I, “Sapienza” University of Rome, Rome, Italy; ^2^Department of Oral and Maxillofacial Sciences, Policlinico Umberto I, “Sapienza” University of Rome, Rome, Italy; ^3^Department of Medical Oncology, Policlinico Umberto I “Sapienza” University of Rome, Rome, Italy

## Abstract

Patients with human papillomavirus- (HPV-) related oropharyngeal squamous cell carcinoma (OPSCC) have a better prognosis than HPV-negative OPSCC when treated with standard high-dose cisplatin-based chemoradiotherapy. Consistent with this assertion and due to younger age at diagnosis, novel approaches to minimize treatment sequelae while preserving survival outcomes become of paramount importance. Here, we critically reviewed the evidence-based literature supporting the deintensification strategies in HPV-related OPSCC management, including radiotherapy dose and/or volume reduction, replacement of cisplatin radiosensitising chemotherapy, and the use of transoral surgery. Undoubtedly, further researches are needed before changing the standard of care in this setting of patients.

## 1. Introduction

Despite oropharyngeal squamous cell carcinoma (OPSCC) representing only 0.9% of all cancer sites, its incidence is rapidly growing worldwide, with an estimated 173,495 new cases in 2018 [1]. The highest incidence rates are seen in the western countries [2]. During the past two decades OPSCC diagnosis increased among men and/or women in different European nations, such as United Kingdom, France, Germany, Denmark, and Sweden [2, 3]. The main reason is oncogenic human papillomavirus (HPV) type 16 infection and nowadays HPV-related OPSCC, primarily located in tonsil and base of tongue, is considered a distinct disease entity [4]. Patients with HPV-related OPSCC have a much better prognosis than those with tobacco/alcohol-driven disease, despite a higher stage at diagnosis due to a typical small primary in the oropharynx with massive regional nodal involvement. Compared with HPV-negative OPSCC, HPV-related OPSCC affects younger patients with a lower comorbidity index, a higher socioeconomic status, and a history of multiple sexual partners and orogenital sexual practice [5]. Intensity modulated radiation therapy with concurrent cisplatin-based chemotherapy represents the standard treatment, when appropriate. This definitive chemoradiotherapy (CRT) approach aims to eradicate tumor cells and minimize both acute and late toxicities. Given the favorable prognosis in a younger patient cohort, novel treatment regimens with the same tumor control and lower toxicity rates are a welcome change.

Here, we presented a critical review of recent advances in the management of HPV-related OPSCC. We focused on the existing literature regarding the proposal applications of radiation therapy and systemic therapy. An assessment of new staging system specifically for HPV-related OPSCC and its development was also reported.

## 2. Materials and Methods

**Figure 1:**
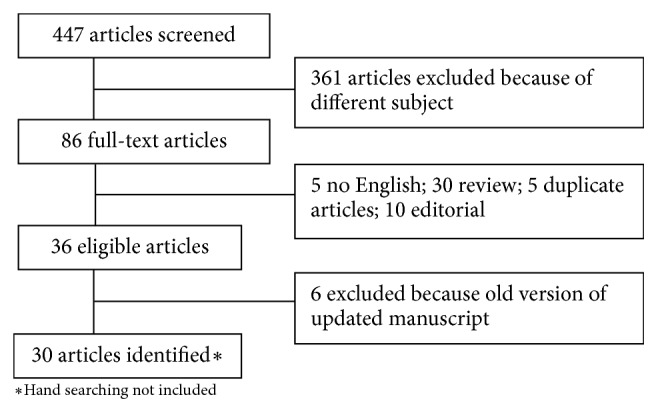
Literature search.

Key HPV-related OPSCC references were derived from a systematic PubMed query. Articles were obtained using the following combinations of research criteria: “intensity modulated radiotherapy”, “imrt”, “radiation therapy”, “de-intensification”, de-escalation”, “immune check-point inhibitors”, “cetuximab”, “cisplatin”, “platinum”, “toxicity”, “quality of life”, “chemotherapy”, “induction”, “treatment”, “transoral surgery”, “tors”, “hpv”, “head and neck cancer”, “oropharyngeal”, “oropharynx” (Figure 1). Hand searching (meeting proceedings of European Society of Medical Oncology, European SocieTy for Radiotherapy & Oncology, American Society for Radiation Oncology and American Society of Clinical Oncology) and clinicaltrials.gov were also used. The last literature search was done in January 2019. Only English written publications were selected. Titles and abstracts of search results were screened to determine eligibility in the manuscript.

## 3. Results

### 3.1. New Classification System for HPV-Related Oropharyngeal Cancer

The 8th edition American joint committee on cancer (AJCC) tumor, lymph node, and metastasis (TNM) staging manual on OPSCC introduced significant modifications from the prior 7th edition [11]. HPV-related OPSCC—based on the overexpression of the cyclin-dependent kinase p16—was part of a separate section. It specifically resulted in a change of T and N categories, due to the important need to discriminate between the different stage groups compared to OPSCC associated to other causes. HPV-related OPSCC clinical (c) T classification no longer included a cT4b category, because 5-year overall survival was similar for patients classed as cT4a and cT4b according to 7th edition TNM staging system [6]. N classification, both clinical and pathological (p), represented the main change from the tobacco/alcohol-driven OPSCC. Because cN1, cN2a, and cN2b (7th edition TNM) cohorts had similar impact on 5-year survival, they were grouped as one cN1 category, including ≥ 1 ipsilateral lymph nodes, none larger than 6 cm whereas cN2c was reserved for contralateral or bilateral lymph nodes, none larger than 6 cm, and cN3 included ≥ 1 lymph nodes larger than 6 cm. The combination of cT and cN into stages—stage I (cT1-2 cN0-1), stage II (cT1-2 cN2 or cT3N0-2), and stage III (cT4 or cN3)—depicted an adequate discrimination in HPV-related OPSCC prognosis groups. Interestingly only distant metastatic disease (M1) was considered stage IV.

The rationale for these changes is based on the international collaboration on oropharyngeal cancer network for staging (ICON-S) multicentre cohort study, including 1907 patients with HPV-related OPSCC from seven institutions across Europe and North America [6]. Several independent external validations have been proposed [7–10]. Details are listed in Table 1. Results showed similar or even better 5-years overall survival rates weighed against the ICON-S study. Globally, these cohorts confirmed that the new classification in HPV-related OPSCC provided better survival discrimination across the different stage categories compared to the 7th edition TNM. Several considerations should be addressed. Firstly, this favorable effect could be mainly driven by the high treatment strategy (surgery and/or CRT). An illustrative example included cT2cN1 disease, now stage I (8th edition TNM) and previously stage III (7th edition TNM). Therefore, it remains unknown whether the high survival rate observed in HPV-related OPSCC patients represents an effective good prognosis factor or merely reflects an overtreatment in this population. Secondly, other factors, such as age, smoke, and alcohol, may potentially even better stratify this setting of patients.

pN categories focused only on number of positive lymph nodes, using a cut-off of 4 to discriminate between pN1 (≤ 4 positive lymph nodes) and pN2 (> 4 positive lymph nodes). Pathologic data emerged from surgical HPV-related OPSCC cohort of 704 patients from five cancer centers [7]. It should be noted that the presence of bilateral/contralateral lymph nodes had prognostic impact (p=0.049) in the univariate analysis for overall survival, as well as extranodal extension (ENE) having a positive trend (p=0.060). An external validation, based on 3745 patients from the national cancer database (NCDB), concluded that ENE could play a prognostic role in HPV-related OPSCC [12]. Results showed a significant negative ENE effect (p < 0.001) on survival. But this effect remained statistically significant when stratified by N-stage only for pN1 disease. Further studies with large cohort of patients are necessary to validate these pathological changes. But, again, maybe, to improve discrimination between pathological groups, more factors, such as bilaterally/contralaterally and ENE, should be considered.

### 3.2. Radiation Therapy in HPV-Related Oropharyngeal Cancer

When appropriate, definitive cisplatin-based CRT using intensity modulated technique (IMRT) is the standard of care in OPSCC. But this approach has drawbacks in terms of toxicity and subsequent patient quality of life (QoL). Considering the good prognostic value of HPV-driven disease, novel treatment paradigms have been proposed in HPV-related OPSCC. These treatment strategies include (i) radiation dose deescalation, (ii) radiation volume deescalation, (iii) induction response-based therapy, (iv) transoral surgery and deintensification of adjuvant treatment. The joint aim is to determine whether a less intensive regimen could minimize toxicity while maintaining similar cure rates.

*Radiation Dose Deescalation.* Late RT-related toxicity represents a significant burden to OPSCC survivors, because it negatively impacts on their QoL and their ability to function in society. The dose delivered to surrounding tissues plays a crucial role in the development of late toxicity. A dose-effect relationship between dose exposure—maximum dose (Dmax) and/or mean dose (Dmean) and/or percentage of volume receiving x Gy (Vx)—of a specific organ at risk (OAR) and development of its related toxicity has been well established. For instance, a Dmean greater than 50 Gy to pharyngeal constrictor muscles, a Dmean greater than 26 Gy to parotid gland, and a V50 greater than 40.5% to mandible can, respectively, cause moderate to severe swallowing impairment, xerostomia, and osteoradionecrosis [13–15]. Ideally each OAR in the head and neck region should receive a low dose exposure to reduce the risk of RT-induced toxicity. But, in OPSCC, RT with curative intent requires large treatment fields and high doses to be effective. Traditionally the total dose delivered to eradicate clinical and subclinical disease is 70 Gy (2 Gy per fraction) and 50 Gy (2 Gy per fraction), respectively. Therefore it is not always feasible to respect all OARs dose constraints, especially for those structures in close proximity to burden tumor, such as dysphagia-related structures, parotid gland, and mandible. Given the IMRT technical ability (that permits including OARs in the optimization process) and the low incidence of regional failures in the elective volume (that receives a prophylactic dose of 50 Gy), deintensification RT strategies could result in toxicity reduction without compromising survival outcomes [16, 17]. Radiation dose deescalation strategies are currently under investigation. A phase III randomized clinical trial was performed to evaluate the dose reduction effect on late toxicity and regional tumor control in head and neck cancer patients [18]. Independently of HPV status, 200 patients with head and neck carcinoma were randomized to the standard dose of 50 Gy versus the experimental dose of 40 Gy prescribed to the elective nodal volumes. Primary end-point was dysphagia at 6 months of follow-up. Results showed a trend to less dysphagia (p = 0.02) and less salivary gland toxicity (p = 0.01) at 6 months without differences in overall, disease-free, and disease-specific survival, as well as local, regional, and distant control. But absolute numbers of regional recurrences and distant metastases were too small to draw definitive conclusions on the safety of dose deescalation to 40 Gy to the elective nodal volume. For sure it represents an interesting approach especially in the context of HPV-related OPSCC, due to the long life expectancy of a patient once his cancer is cured.

A parallel between HPV-related OPSCC and HPV-related anal canal carcinoma could be even more interesting. In fact, these two malignancies presented similar tumor histology and viral etiology. A main consideration can be made in the context of organ preservation strategy, using combined CRT modality. In anal canal carcinoma, a total dose of 59.4 Gy (1.8 Gy per fraction) with concurrent chemotherapy is recommended to assure a curative intent [19]. Therefore it could be reasonable to prescribe a lower radiation dose (≤ 60 Gy) plus concomitant chemotherapy in the treatment of HPV-related OPSCC. It might result in similar clinical outcomes decreasing toxicity rates. Evidence is accumulating that radiation dose deescalation can refer to primary tumor target volume [20]. In a phase II trial, 43 favorable risk HPV-related OPSCC patients were treated with IMRT to a total dose of 60 Gy (2 Gy per fraction) plus concomitant weekly cisplatin (30 mg/m^2^ per week). Compared to standard CRT regimen, radiation dose was reduced by 16% (70 to 60 Gy) and cumulative chemotherapy dosage was reduced by 60% (300 mg/m^2^ to 180 mg/m^2^). Primary end-point was pathological complete response (pCR) based on biopsy of the primary site and a limited or selective neck dissection of pretreatment positive lymph node regions. This allowed for a more patient safety standpoint due to authors being worried for detrimental outcomes of deintensified strategy. The pCR rate was 86% with relatively decreased toxicity. Globally results were encouraging, but a randomized clinical trial to make a direct comparison to standard regimen is paramount to assess the real impact of deintensified CRT on both long-term tumor control and toxicities.

Recently, the Memorial Sloan-Kettering Cancer Center group performed a pilot study to test hypoxia imaging—^18^F-fluorodeoxyglucose (^18^F-FDG) and dynamic ^18^F-fluoromisonidazole (^18^F-FMISO) positron emission tomography (PET)—as selection criteria for radiation dose deescalation to gross nodal disease in HPV-related OPSCC patients [21]. Stages III-IVb HPV-related OPSCC (7th edition) patients without pretreatment hypoxia or with resolution of hypoxia within 1 week of treatment on intratreatment ^18^F-FMISO PET received a 10 Gy dose reduction (from 70 Gy to 60 Gy) to either the primary site and/or lymph node(s). Of the 33 patients enrolled, 10 patients (30%) met the criteria for radiation dose deescalation. At a median follow-up of 32 months, the 2-year locoregional control, overall survival, and distant metastasis-free survival were 100%, 100%, and 97%, respectively, with minimal toxicity. This approach emphasized the potential role of ^18^F-FMISO PET to guide therapeutic decisions, but further studies are necessary.

Several studies attested the high radiosensitivity of HPV-related OPSCC, reporting comparable clinical outcomes in patients with HPV-positive head and neck cancer treated with definitive RT alone instead of standard CRT [22–24]. The reported influence of tumor HPV-status on RT responsiveness should be considered in radiation dose deescalation strategies, even though exactly how to individualized treatment remains uncertain. In this context, the NRG Oncology cooperative group is leading a randomized phase II trial (NCT02254278) to test exclusive modestly reduced-dose IMRT (60 Gy, 2.4 Gy per fraction) versus CRT (weekly 40 mg/m^2^ cisplatin and 60 Gy, 2 Gy per fraction) in 296 planned patients with cT1-2, cN1-2b, or cT3, N0-2b (7th edition) HPV-related OPSCC and a lifetime cumulative smoking history < 10 pack-years [25].

*Radiation Volume Deescalation.* Several investigators assumed that limited radiation to the ipsilateral neck without compromising locoregional control could be feasible in selected patients also in the HPV era [26–30]. In general, elective neck irradiation is not recommended if subclinical disease risk is < 10%, due to RT morbidity [29]. Compared to bilateral irradiation, unilateral neck irradiation permitted to better spare OARs and reduce the risk of RT-related side effects, such as xerostomia, improving patients' QoL [26]. A recent publication showed that ipsilateral RT continued to be safe and contralateral neck failure remained low for patients with cT1-2 cN0-2b (7th edition) HPV-related tonsillar cancer [30]. With regard to control of lymphatic spread, a careful case selection—well-lateralized lesion, without extension to soft palate or tongue base, without muscle involvement or any suspicion of deeper penetration, and no contralateral neck lymph node metastasis— become essential. Prospective clinical trials addressing the suitability of ipsilateral radiation in HPV-related OPSCC are warranted to confirm the efficacy of this approach. Restaging of HPV-related OPSCC series according to 8th edition TNM and reevaluation of previous treatment indications could result in a change of the therapeutic strategies for HPV-related OPSCC. Probably radiation volume deescalation is only imaginable in low-risk HPV-positive patients.

*Induction Response-Based Therapy. *Different groups have pursued an approach of radiation dose deescalation following the use of induction chemotherapy [31–35].

The Optima trial was a phase II deescalation study designed for patients with HPV-related OPSCC [31]. Induction chemotherapy was adopted to identify favorable patients to apply significantly lower (chemo) radiation doses than standard CRT. Patients were classified as low-risk (≤ T3, ≤ N2b, ≤ 10 pack-year history) and high-risk (T4 or ≥ N2c or >10 pack-year history). They received induction chemotherapy, including 3 cycles of carboplatin (AUC 6) and nab-paclitaxel (100 mg/m^2^). Based on response to induction treatment, locoregional therapy was stratified as (i) low-dose RT alone to 50 Gy (2 Gy per fraction) in low-risk patients with ≥ 50% response, (ii) low-dose CRT to 45 Gy (1.5 Gy twice-daily fraction and paclitaxel, 5-fluorouracil, and hydroxyurea) in low-risk patients with 30-50% response or high-risk patients with ≥ 50% response, (iii) standard-dose CRT to 75 Gy (1.5 Gy twice-daily fraction and paclitaxel, 5-fluorouracil, and hydroxyurea) in poor responders. Primary site biopsy and neck dissection were performed only after deescalated (C)RT for pathologic confirmation. The primary endpoint was 2-year progression-free survival (2-y PFS). With a median follow-up of 29 months, the 62 patients enrolled achieved excellent 2-y PFS rates (95% for low risk patients, 94% for high risk patients). Severe acute toxicity, including oral mucositis, skin dermatitis, and PEG-tube requirement, was significantly lower with deescalated treatment. These results compare favorably to the historical control and justified the evaluation of this strategy in a larger comparative trial. But it should be noticed that standard-dose CRT scheme—1.5 Gy twice-daily fraction and paclitaxel, 5-fluorouracil, and hydroxyurea—differed from the standard of care cisplatin-based CRT treatment.

Similarly, the ECOG-ACRIN Cancer Research Group trial evaluated induction chemotherapy (cisplatin, paclitaxel, and cetuximab) followed by concurrent cetuximab and RT to 54 Gy (2 Gy per fraction), complete responders, or 69.3 Gy (2.1 Gy per fraction), no-complete responders, in HPV-related OPSCC patients [32]. The primary end-point was 2-y PFS. Globally, 80 patients were evaluated. After a median follow-up of 35.4 months, 2-y PFS was 80% in cohort with clinical complete response. Interestingly, treatment failures occurred within 2 years after accrual and were recorded on patients with a > 10 pack-year smoking history. Significantly fewer patients treated with dose deescalation had difficulty swallowing solids or impaired nutrition.

Another ongoing US single-arm phase II trial investigated whether weekly paclitaxel CRT with radiation dose deescalation would maintain survival outcomes while improving functional outcomes [33]. After two cycles of paclitaxel/carboplatin-based induction chemotherapy, complete or partial responders received 54 Gy (2 Gy per fraction) and those with less than partial or no responses received 60 Gy (2 Gy per fraction). The primary endpoint was 2-y PFS. A total of 45 patients with stages III-IV (7th edition) HPV-related OPSCC were enrolled. Median follow-up was 30 months and 2-y PFS rate was 92% with an acceptable toxicity profile.

The Quarterback is an active phase III trial that directly compared a radiation dose deescalation to the standard of care in HPV-positive patients [34]. After 3 cycles of docetaxel cisplatin and 5-fluorouracil induction chemotherapy, patients with a clinical or radiographic complete/partial response are randomized to receive a reduced (56 Gy) or standard (70 Gy) dose RT with weekly carboplatin. A total of 365 patients with advanced HPV-related oropharynx cancer, nasopharynx cancer, or unknown primary are planned to determine the comparative rate of PFS at 3 years. Preliminary results—based on 23 patients enrolled and 20 randomized–have been presented at ASCO meeting in 2017 and the 2-y PFS rates were 87.5% for those patients receiving standard dose and 83.3% for those patients receiving dose deescalation [35].

Globally, all these studies indicated that HPV-related OPSCC could be successfully treated with a sequential treatment strategy of induction chemotherapy followed by radiation dose deescalation preserving both clinical and functional outcomes. Definitive phase III randomized clinical trials adopting the 8th edition TNM classification and standard of care treatment arm are paramount to confirm these results, define appropriate candidates, and alter standard clinical practice. Surely, independently of radiation treatment modalities—dose deescalation, volume deescalation, and following induction chemotherapy—the high-quality RT is paramount to guarantee reliable treatment outcome.

*Transoral Surgery and Deintensification of Adjuvant Treatment.* Adjuvant (C)RT dose reduction following primary transoral surgery is also being proposed as an alternative deescalation treatment strategy for HPV-related OPSCC. Its main advantage is the proper adjuvant treatment based on objective criteria driven by pathologic staging. To our knowledge, there are as yet no published prospective randomized data on this topic, but several clinical trials are ongoing [36–41]. Actually, the Mayo Clinic group presented at ASTRO 2017 meeting the results of the phase II MC1273 trial but full-text is still not available [36]. This study included patients with HPV-related OPSCC and ≤ 10 pack-year smoking history. Following surgery with negative margins, patients with ≥T3, ≥N2, lymphovascular invasion, or perineural invasion received 30 Gy (1.5 Gy twice daily fraction) with concomitant docetaxel. In case of evidence of extracapsular spread, patients received the same treatment plus a simultaneous integrated boost to nodal levels with extracapsular spread to 36 Gy (1.8 Gy twice daily fraction). Results showed a locoregional control rate (95%) comparable to historical controls. No patients required feeding tube. Based on these data, a phase III multicenter study (DART-HPV trial) has been designed and is actively accruing [37]. A total of 214 are planned. Patients are randomized to receive deescalated adjuvant docetaxel-based CRT (30 Gy in 1.5 Gy fractions twice daily in intermediate risk patients or 36 Gy in 1.8 Gy fractions twice daily in high risk patients) versus standard of care treatment with weekly cisplatin 40 mg/m^2^ concomitant to RT to 60 Gy delivered in 2 Gy per fraction. Primary end-point is adverse events rate at 2 years.

The ECOG-ACRIN Cancer Research Group designed a phase II trial for stages III-IVb HPV-related OPSCC [38]. cN0 patients are not eligible. Based on their risk status —low risk: no adverse pathological features, intermediate risk: T1-3, N2a-2b, perineural and/or vascular invasion or close margins, and high risk: positive margins and/or extracapsular spread—patients are assigned to (i) transoral robotic surgery (TORS) alone (low risk), (ii) TORS and low-dose RT, 50 Gy 2 Gy per fraction (intermediate risk), (iii) TORS and standard dose RT, 60 Gy 2 Gy per fraction (intermediate risk), and (iv) TORS and standard dose weekly platinum-based CRT, 66 Gy 2 Gy per fraction (high risk). Patients classified as intermediate risk are randomized to low-dose or standard dose treatment arm. Primary end-point is 2-y PFS.

In the ADEPT trial, HPV-related OPSCC patients received either RT alone (60 Gy, 2 Gy per fraction) or weekly cisplatinum-based CRT (60 Gy, 2 Gy per fraction) after margin-clearing TORS of their T1-4a oropharynx primary (7th edition) and a neck dissection with extracapsular spread in their lymph nodes [39]. Primary end-points were 5-year disease-free survival and 5-year locoregional control.

The primary outcome of the prospective randomized PATHOS study is to improve patient-reported swallowing outcome testing adjuvant dose deescalation RT in order to continue to a phase III noninferiority study with overall survival as the primary end-point [40]. Patients with stage T1-3, N0-2b (7th edition) HPV-related OPSCC, are enrolled. Following surgery and based on pathological risk factors for recurrence, patients will receive (i) no adjuvant treatment, (ii) randomization to adjuvant RT to 60 Gy (2 Gy per fraction) or 50 Gy (2 Gy per fraction), and (iii) randomization to adjuvant weekly cisplatin-based CRT to 60 Gy (2 Gy per fraction) or RT alone to 60 Gy (2 Gy per fraction).

An interesting approach was proposed by the Memorial Sloan Kettering Cancer Center [41]. Investigators conducted a pilot study using ^18^F- FMISO PET to identify HPV-related OPSCC patients eligible for adjuvant dose deescalation. Patients received surgery to primary tumor only, whereas lymph nodes were evaluated by ^18^F- FMISO PET. Patients without hypoxia or with resolution at intratreatment ^18^F- FMISO PET received 30 Gy (2 Gy per fraction) to the tumor bed and neck with 2 cycles of concurrent high-dose cisplatin or carboplatin/5-FU. Patients with persistent hypoxia received standard CRT up to 70 Gy. Neck dissection was performed 4 months after CRT. In total 19 patients were enrolled and 15 patients were deescalated to 30 Gy. Globally, 18 out of 19 patients (95%) remain disease free. A multicenter trial to validate these pilot results is ongoing.

In summary, waiting for definitive results of the proposed trials, no firm conclusions can be drawn. We agree with the principle of pathological risk and functional imaging assessment to guide treatment deescalation decisions.

### 3.3. Systemic Therapy in HPV-Related Oropharyngeal Cancer

Efforts to minimize acute and late toxicity of primary CRT in HPV-related OPSCC patients also include systemic therapy. The options are (i) replacing cisplatin with the epidermal growth factor receptor (EGFR) inhibitor cetuximab and (ii) replacing cisplatin with immune check-point inhibitors.

*Replace Cisplatin with Cetuximab.* Cetuximab is an IgG1 monoclonal antibody against the EGFR approved by the US Food and Drug Administration in 2006 due to its proven survival benefit (median survival from 29.3 months to 49 months) without increasing the common toxic effects compared to RT alone in locally advanced head and neck cancer (IMCL-9815 trial) [42]. The updated data of IMCL-9815 trial for subgroup analyses of patient and tumor factors suggested a potential increased survival benefit from cetuximab in those patients with early T stage and advanced N stage OPSCC, age < 65 years, and high performance status [43]. Importantly, the IMCL-9815 trial was not powered for this subgroup analysis. Therefore these data could be ascribable to chance, but it should be noted that these characteristics are common to patients with HPV-related disease and this finding has encouraged research groups to test the use of cetuximab in these patients. Two randomized noninferiority trials, the De-ESCALaTE HPV trial and the RTOG 1016 trial, proposed cetuximab for treatment deescalation strategy in HPV-related OPSCC [44, 45]. The aim was to reduce standard cisplatin-based CRT toxicity profile while preserving survival efficacy. Final data analyses were published online in November 2018. Contrary to expectations, replacing cisplatin with cetuximab demonstrated a significantly detrimental impact on survival end-points, in both trials. In light of these results, RT plus cetuximab cannot be considered a deescalation strategy to reduce toxicity while maintaining survival in patients with HPV-related OPSCC. Cisplatin-based CRT remains the standard of care.

*Replace Cisplatin with Immune Check-Point Inhibitors.* During the past few years, there has been an exciting development of immunotherapy, especially check-point inhibitors in different human malignancies, including head and neck cancer [46]. The immune check-point inhibitors represent a successful immunotherapeutic approach, due to their peculiar ability to target lymphocyte receptors, as opposed to target therapy, such as cetuximab, that act directly on the tumor cells [47]. They mainly include antiprogrammed death-1 (PD-1) antibody and anticytotoxic T lymphocyte associated antigen 4 (CTLA-4) antibody. Nivolumab and pembrolizumab are both anti-PD-1 antibody and are recommended as categories 1 and 2a, respectively, in recurrent and/or metastatic head and neck cancer (nonnasopharyngeal cancer) if disease progresses on or after platinum-based chemotherapy [4]. Based on phase III CheckMate 141 study (nivolumab) and phase Ib KEYNOTE-012 trial (pembrolizumab), deintensification by replacing cisplatin with immune check-point inhibitors could represent a promising strategy to achieve optimum disease control with minimal long-term toxicities in HPV-related OPSCC with favorable risk disease.

A phase II study with safety lead-in has been designed to test safety, tolerability, and efficacy of anti-CTLA4 (ipilimumab) and anti-PD-1 (nivolumab) in combination with RT up to 60 Gy (2 Gy per fraction) in patients with 8th edition stages T1N2, T2N1-2, and T3N0-2 HPV-related OPSCC [48]. This study is not yet recruiting.

*[To note, the potential role of RT combination with these agents has recently been proposed in patients with HPV-related OPSCC with smoking status > 10 pack-years, stage T1-2N2b-N3, or ≤ 10 pack-years, stages T4N0-N3 or T1-3N3 [49]. The aim is to test the safety of nivolumab added to several CRT regimens, including weekly cisplatin, high-dose cisplatin, cetuximab, or IMRT alone. Final data collection for primary outcome measures is estimated in March 2019.]*

Enrolment in current trials of RT plus immune check-point inhibitors in this patient population should be strongly encouraged where possible.

## 4. Conclusions

**Figure 2:**
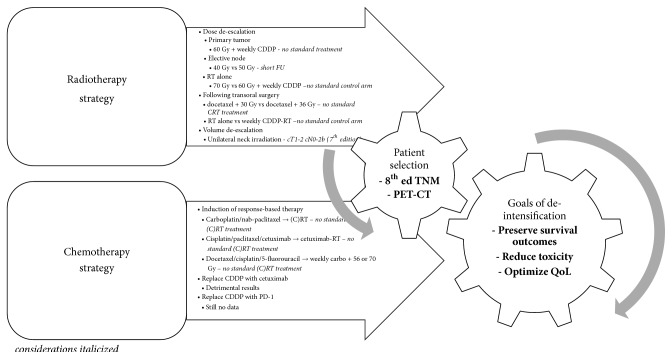
Deintensification strategies.

At present, HPV-related OPSCC can be considered a distinct disease primarily as a consequence of its anatomical location and its viral aetiology. Its optimal treatment approach is still not well-defined. For sure, HPV-related OPSCC is extremely sensitive to radiation exposure and patients generally are complete responders and long-term survivors. Therefore over the years scientific interest has shifted to new stratagems to potentially improve functional outcomes.  Figure 2 summarizes the main deintensification strategies, based upon the published literature discussed above. We believe that Figure 2 could add value to the indirect comparisons of these methods. It must be appreciated that its bullet points are suggestions to standardize protocols and develop a gold-standard assessment panel. In fact, an important question is how to best implement both intradisciplinary and interdisciplinary into the current HPV-related OPSCC management. Actually, the vast majority of clinical trial is testing different approaches. Thus, in the coming years, there will be a big data disorder that could delay the expected change in the standard of care. It should emphasize the importance of a trial design and the value to compare what is already conformed to the standard. In addition, accurate patient selection should be critical to optimal implementation of a new strategy. Research groups should endeavor to consider such observations to implement and optimize clinical results. At present no changes in HPV-related OPSCC management should be made outside clinical trials.

## Figures and Tables

**Table 1:** Independent external validation of the 8th edition staging of HPV-related oropharyngeal cancer.

				5-y OS by 8th edition TNM stage			
Author	Year of publication	Patients	Primary treatment	I	II	III	IV
*O'Sullivan [6]*	*2016*	*1907*	*S: 34; RT: 1873*	*85%*	*78%*	*53%*	*NA*

Haughey [7]	2016	704	S: 704	90%	84%	48%	NA

Cramer [8]	2017	15116	S: 6465; RT: 7841; CHT: 276	87.4%*∗*	76.6%*∗*	63.1%*∗*	20.7%*∗*

Malm [9]	2017	435	S: 166; RT: 269	92.3%	87.2%	73.6%	40.0%

Porceddu [10]	2017	279	RT: 279	93.6%	81.9%	69.1%	NA

international collaboration on oropharyngeal cancer network for staging (ICON-S) study

*∗*4-year overall survival

5-y OS: 5-year overall survival; S: surgery; RT: radiotherapy; CHT: chemotherapy; NA: not applicable
